# *BRAF* ^V600E^/*RAS* Mutations and Lynch Syndrome in Patients With MSI-H/dMMR Metastatic Colorectal Cancer Treated With Immune Checkpoint Inhibitors

**DOI:** 10.1093/oncolo/oyad082

**Published:** 2023-04-06

**Authors:** Raphael Colle, Sara Lonardi, Marine Cachanado, Michael J Overman, Elena Elez, Marwan Fakih, Francesca Corti, Priya Jayachandran, Magali Svrcek, Antoine Dardenne, Baptiste Cervantes, Alex Duval, Romain Cohen, Filippo Pietrantonio, Thierry André

**Affiliations:** Sorbonne University, Department of Medical Oncology, Saint-Antoine Hospital, AP-HP, Paris, France; Sorbonne University, SIRIC CURAMUS, INSERM, Unité Mixte de Recherche Scientifique 938, Centre de Recherche Saint-Antoine, Equipe Instabilité des Microsatellites et Cancer, Equipe Labellisée par la Ligue Nationale Contre le Cancer, Paris, France; Sorbonne University, Department of Clinical Pharmacology and Clinical Research Platform Paris-East (URCEST-CRC-CRB), Assistance Publique-Hôpitaux de Paris, Sorbonne University, St Antoine Hospital, Paris, France; Oncology Department, Istituto Oncologico Veneto IOV-IRCSS, Padua, Italy; Sorbonne University, Department of Clinical Pharmacology and Clinical Research Platform Paris-East (URCEST-CRC-CRB), Assistance Publique-Hôpitaux de Paris, Sorbonne University, St Antoine Hospital, Paris, France; Department of Gastrointestinal Oncology, University of Texas MD Anderson Cancer Center, Houston, TX, USA; Department of Medical Oncology, Vall d’Hebron Barcelona Hospital Campus, Vall d’Hebron Institute of Oncology (VHIO), Universitat Autonoma de Barcelona, Barcelona, Spain; Department of Medical Oncology and Therapeutic Research, City of Hope Comprehensive Cancer Center, Duarte, CA, USA; Department of Medical Oncology, Fondazione IRCCS Istituto Nazionale dei Tumori, Milan, Italy; Division of Medical Oncology, Norris Comprehensive Cancer Center, Keck School of Medicine, University of Southern California, Los Angeles, CA, USA; Sorbonne University, SIRIC CURAMUS, INSERM, Unité Mixte de Recherche Scientifique 938, Centre de Recherche Saint-Antoine, Equipe Instabilité des Microsatellites et Cancer, Equipe Labellisée par la Ligue Nationale Contre le Cancer, Paris, France; Sorbonne University, Department of Pathology, Saint-Antoine Hospital, AP-HP, Paris, France; Sorbonne University, Department of Medical Oncology, Saint-Antoine Hospital, AP-HP, Paris, France; Sorbonne University, Department of Medical Oncology, Saint-Antoine Hospital, AP-HP, Paris, France; Sorbonne University, SIRIC CURAMUS, INSERM, Unité Mixte de Recherche Scientifique 938, Centre de Recherche Saint-Antoine, Equipe Instabilité des Microsatellites et Cancer, Equipe Labellisée par la Ligue Nationale Contre le Cancer, Paris, France; Sorbonne University, Department of Clinical Pharmacology and Clinical Research Platform Paris-East (URCEST-CRC-CRB), Assistance Publique-Hôpitaux de Paris, Sorbonne University, St Antoine Hospital, Paris, France; Sorbonne University, Department of Medical Oncology, Saint-Antoine Hospital, AP-HP, Paris, France; Sorbonne University, SIRIC CURAMUS, INSERM, Unité Mixte de Recherche Scientifique 938, Centre de Recherche Saint-Antoine, Equipe Instabilité des Microsatellites et Cancer, Equipe Labellisée par la Ligue Nationale Contre le Cancer, Paris, France; Sorbonne University, Department of Clinical Pharmacology and Clinical Research Platform Paris-East (URCEST-CRC-CRB), Assistance Publique-Hôpitaux de Paris, Sorbonne University, St Antoine Hospital, Paris, France; Department of Medical Oncology, Fondazione IRCCS Istituto Nazionale dei Tumori, Milan, Italy; Sorbonne University, Department of Medical Oncology, Saint-Antoine Hospital, AP-HP, Paris, France; Sorbonne University, SIRIC CURAMUS, INSERM, Unité Mixte de Recherche Scientifique 938, Centre de Recherche Saint-Antoine, Equipe Instabilité des Microsatellites et Cancer, Equipe Labellisée par la Ligue Nationale Contre le Cancer, Paris, France; Sorbonne University, Department of Clinical Pharmacology and Clinical Research Platform Paris-East (URCEST-CRC-CRB), Assistance Publique-Hôpitaux de Paris, Sorbonne University, St Antoine Hospital, Paris, France

**Keywords:** deficient mismatch repair, metastatic colorectal cancer, immune checkpoint inhibitors, Lynch syndrome, *RAS* mutation, *BRAF* mutation

## Abstract

**Background:**

We pooled data from 2 cohorts of immune checkpoint inhibitors-treated microsatellite instability-high/mismatch repair-deficient (MSI/dMMR) metastatic colorectal cancer patients to evaluate the prognostic value of *RAS*/*BRAF*^V600E^ mutations and Lynch syndrome (LS).

**Patients and Methods:**

Patients were defined as LS-linked if germline mutation was detected and as sporadic if loss of MLH1/PMS2 expression with *BRAF*^*V600E*^ mutation and/or *MLH1* promoter hypermethylation, or biallelic somatic MMR genes mutations were found. Progression-free survival (PFS) and overall survival (OS) were adjusted on prognostic modifiers selected on unadjusted analysis (*P* < .2) if limited number of events.

**Results:**

Of 466 included patients, 305 (65.4%) and 161 (34.5%) received, respectively, anti-PD1 alone and anti-PD1+anti-CTLA4 in the total population, 111 (24.0%) were treated in first-line; 129 (28.8%) were *BRAF*^*V600E*^-mutated and 153 (32.8%) *RAS*-mutated. Median follow-up was 20.9 months. In adjusted analysis of the whole population (PFS/OS events = 186/133), no associations with PFS and OS were observed for *BRAF*^*V600E*^-mutated (PFS HR= 1.20, *P* = .372; OS HR = 1.06, *P* = .811) and *RAS*-mutated patients (PFS HR = 0.93, *P* = .712, OS HR = 0.75, *P* = .202). In adjusted analysis in the Lynch/sporadic status-assigned population (*n* = 242; PFS/OS events = 80/54), LS-liked patients had an improved PFS compared to sporadic cases (HR = 0.49, *P* = .036). The adjusted HR for OS was 0.56 with no significance (*P* = .143). No adjustment on *BRAF*^*V600E*^ mutation was done due to collinearity.

**Conclusion:**

In this cohort, *RAS/BRAF*^*V600E*^ mutations were not associated with survival while LS conferred an improved PFS.

## Teaser

Using data from 2 cohorts of patients with MSI/dMMR metastatic colorectal cancer treated with immune checkpoint inhibitors, this study evaluated the prognostic value of RAS/BRAFV600E mutations and Lynch syndrome.

**Implications for Practice**

Patients with MSI-H/dMMR metastatic colorectal cancer (mCRC) treated with immune checkpoint inhibitors had impressive survival results. In this population, *RAS and BRAF*^*V600E*^ mutations in tumor are not prognostic factors for progression-free survival (PFS) and overall survival. In the absence of a standardized definition, an algorithm presented in this study based on immunochemistry and molecular data, define patients with MSI-H/dMMR mCRC, Lynch syndrome, and sporadic cases. In this population, patients with Lynch syndrome had better PFS compared with those with sporadic cases.

## Introduction

Immune checkpoint inhibitors (ICIs) have revolutionized the treatment and prognosis of microsatellite instability-high/mismatch repair-deficient (MSI-H/dMMR) metastatic colorectal cancer (mCRC). Several phase II trials showed that anti- programmed cell death-1 (PD1) either as monotherapy or in combination with anti-cytotoxic T-lymphocyte-associated antigen-4 (CTLA4) exhibited high efficacy and survival benefit in MSI-H/dMMR mCRC.^1-3^ The phase III KEYNOTE-177 study demonstrated superiority for first-line pembrolizumab over chemotherapy in terms of median progression-free survival (PFS) of 16.5 vs. 8.2 months; hazard ratio (HR) = 0.60, 95% CI, 0.45-0.80, *P* = .0002).^4^ The KEYNOTE-177 study did not report a statistically significant overall survival (OS) benefit with pembrolizumab vs. chemotherapy per the published statistical plan; this was likely due to high rate (60%) of crossover to ICIs in the chemotherapy arm after progression.^5^ Based on these data, pembrolizumab was approved by the Food and Drug Administration and European Medicines Agency. Despite high rates of response and a clinical benefit with ICIs, 20%-31% of patients with MSI-H/dMMR mCRC experience primary resistance, frequently resulting in delaying other effective therapies, autoimmune toxicities, and significant collective cost.^1,3,4^ Thus, it is crucial to identify a subpopulation of MSI-H/dMMR mCRC patients with primary resistance to ICIs.

Molecular heterogeneity of MSI-H/dMMR mCRC probably impairs prognosis and the efficacy of ICIs. Notably, the role of *RAS/BRAF*^V600E^ mutational status and the origin of DNA MMR system (Lynch syndrome vs. sporadic CRC) in the efficacy of ICIs for MSI-H/dMMR mCRC is  uncertain.^2-5^ For stage III colon patients with cancer receiving adjuvant FOLFOX, *BRAF*, or *KRAS* mutations are independently associated with shorter survival in those with microsatellite-stable colon cancer, but not MSI tumors.^6,7^ In mCRC, *RAS/BRAF*^*V600E*^ mutations are well-known molecular modifiers of prognosis with an impact on anti-cancer therapies such as anti-EGFR targeted strategies. The results of an analysis of PFS in pre-specified subgroup of *RAS* mutated MSI-H/dMMR mCRC in the KEYNOTE 177 study call into question the superiority of pembrolizumab in this subpopulation (HR = 1.14, 95% CI, 0.68-2.07). Yet, *RAS* mutational status data were lacking in 30% of patients and this lack of effect was less apparent in the OS subgroup analysis (HR = 0.92, 95% CI, 0.48-1.75).^4,5^

**Figure 1.**
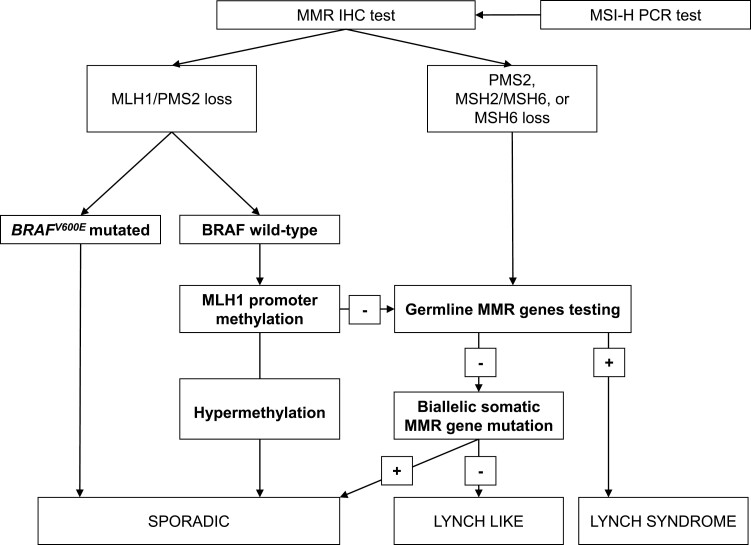
The algorithm for the Lynch syndrome classification according to the order of execution of MSI PCR test and MMR IHC test. IHC, immunohistochemistry; MMR, mismatch repair gene; MSI-H, microsatellite instability-high; PCR, polymerase chain reaction.

It is known that Lynch-associated CRCs (germline mutations in MMR genes [*MLH1*, *PMS2*, *MSH2*, *MSH6*, *EPCAM*]) have distinct pathway of tumorigenesis and clinicopathologic features that sporadic tumors (*MLH1* promoter hypermethylation or biallelic somatic mutations).^8-10^ Several studies have shown that Lynch-associated CRC or endometrial cancer generally presents with more pronounced local T-cell infiltration and even a higher mutational burden compared with sporadic MSI-H CRC, which can support different responses to ICIs.^11,12^ Published data from clinical trials of MSI-H CRC have not shown any significant difference in the efficacy among patients with known Lynch syndrome.^1-3^ However, these trials lacked rigorous criteria for distinguishing Lynch-related tumors from sporadic. In fact, only proven germline MMR gene mutation should confirm Lynch-associated CRC. Whereas those with loss of MLH1/PMS2 expression associated with *MLH1* promoter hypermethylation or *BRAF*^*V600E*^ mutation and with biallelic somatic mutations of MMR genes should be classified as sporadic (Fig. 1).

Here we evaluate the impact of *RAS*/*BRAF*^V600E^ mutational status and Lynch syndrome on prognosis of patients with MSI-H/dMMR mCRC treated with ICIs.

## Methods

### Patients

In this international multicenter study, we analyzed data from 2 pre-existing prospective cohorts of MSI-H/dMMR mCRC patients who received anti-PD1 monotherapy or the anti-PD1 plus anti-CTLA4 combination. The first immuno-MSI French cohort included all consecutive MSI-H/dMMR mCRC patients treated at Saint-Antoine Hospital (Paris, France) from February 2015 to December 2021. This cohort was approved by the ethics committee (N°2020-CER 2020-6). The second multicentric international cohort included MSI-H/dMMR patients with mCRC treated at centers in Italy, Spain, and the United States between November 2014 and November 2021. Ethical approval for the second cohort was provided by the Institutional Review Board of Fondazione IRCCS Instituto Nazionale dei Tumori of Milan (INT 117/15).

### Molecular Data

MSI-H/dMMR status was determined by immunohistochemistry and/or multiplex polymerase chain reaction. *RAS* (*KRAS* and *NRAS*)/*BRAF*^V600E^ mutational status, *MLH1* promoter hypermethylation status, and MMR germline mutations testing were done using local practice according to international guidelines.

We developed Lynch/sporadic classification algorithm by interrogating available immunochemistry and molecular data. Patients were considered to have Lynch syndrome-associated CRC only in case of determined germline mutation and were deemed to have sporadic CRC only if loss of MLH1/PMS2 protein expression associated with *BRAF*^V600E^ mutation and/or hypermethylation of *MLH1* promoter or biallelic somatic mutations of MMR genes (Fig. 1).

### Radiological Analyses

Tumor radiological assessment was done ≤28 days before the first dose (baseline) of ICI and every 6-10 weeks, thereafter, according to different protocols. The radiological response was evaluated by the Response Evaluation Criteria in Solid Tumors (RECIST) 1.1 criteria with the possibility to pursue treatment beyond initial RECIST 1.1-defined progression at the treating physician’s discretion in case of clinical benefit. In this multicenter cohort, scans were not reviewed centrally for the purposes of this study. When a pseudoprogression was suspected, treatment beyond RECIST 1.1 progressive disease was conditional to a locally confirmatory imaging done at 4-8 weeks after the first evidence of progression. In this case, confirmed primary progression was defined according to immune RECIST (iRECIST) criteria and imaging was retrospectively and locally reviewed by an experienced radiologist according to RECIST 1.1 and iRECIST (confirmed progressive disease).^13^

### Statistical Analysis

Categorical variables were described by numbers and percentages and continuous variables by means, SDs, and minimum and maximum values.

The primary endpoint was PFS defined as time from the first injection of ICIs to the first disease progression per iRECIST or death from any cause. Secondary endpoints were OS, defined as time from the first injection of ICIs to death, whatever the cause and overall response rate, defined as the proportion of patients achieving partial or complete response according to iRECIST criteria. Survival curves were generated using the Kaplan-Meier method. Cox regression models were used to compare OS and PFS between groups. The results were expressed as hazard ratios with 95% CIs. The following risk factors were studied: *BRAF*^*V600E*^, *RAS* mutational status Lynch syndrome, age at start of ICI therapy, sex, sidedness (left vs. right), treatment type (anti-PD1 vs. anti-PD1 plus anti-CTL4), Eastern Oncology Cooperative Group performance score (ECOG PS; 0 vs. 1 vs. 2), primary tumor surgery (yes vs. no), number of metastatic sites (≥ 2 vs. 1), ICIs used in first-line (yes vs. no). The number of variables selected for adjusted analysis was limited to 5 events per variable and selected with a *P*-value of < .20 in unadjusted analysis if necessary. Center was considered as stratification variable. Risk proportionality hypothesis was checked for all variables and for continuous variable, log linearity hypothesis was checked. *BRAF*^V600E^ and Lynch syndrome were analyzed separately due to collinearity. Models were performed in patients with available data for all studied variables.

Given the molecular precision required for classification of patient according to our algorithm, a large number of patients with an indeterminate Lynch syndrome or sporadic was expected, therefore we planned to perform 2 analyses including (1) patients with known *RAS*/*BRAF*^V600E^ status and (2) patients with known Lynch syndrome/sporadic status. A log-rank test was used to compare PFS between groups according to germline mutation in Lynch patients. All superiority tests were 2-sided and *P*-values of <.05 were considered significant. Statistical analyses were performed using SAS software (version 9.4; SAS Institute Inc., Cary, NC).

## Results

### Population

**Figure 2.**
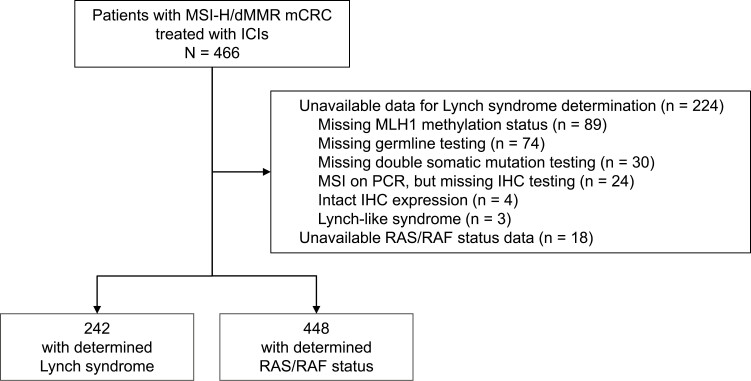
Flow chart of the study with reasons for Lynch and sporadic indeterminations in excluded patients.

A total of 466 MSI-H/dMMR patients with mCRC treated with ICIs were included (Fig. 2); 448 (96.1%) had known *RAS*/ *BRAF*^V600E^ status, 111 (24.0%) received ICIs in first-line, 305 (65.4%) received anti-PD1 alone, and 161 (34.5%) the anti-PD1 plus anti-CTLA4 combination. The prevalence of *BRAF*^*V600E*^ mutation was 28.8% (129/448), *RAS* mutation was 34.1% (153/448), and of *RAS*/*BRAF*^V600E^ wild type was 37.1% (166/448). Baseline characteristics of the patients in the whole study cohort are shown in Table 1. In total, 118 (25.3%) patients were diagnosed with Lynch syndrome and 124 (26.6%) with sporadic CRC (Fig. 2). Table 2 shows characteristics of these 2 groups. Compared with sporadic CRC, patients with Lynch syndrome were younger (49.8 vs. 66.4 years), were often male (67.8% vs. 41,1%), had fewer right-sided tumors (61.8% vs. 2.3%), had higher prevalence of *RAS* mutation (46.6% vs. 8.1%), and lower prevalence of *BRAF*^*V600E*^ mutation (0.8% vs. 75.8%). There were no differences in main clinical characteristics (age, sex, number of prior chemotherapy lines, number of metastatic sites, and ECOG PS) between patients with undetermined germline mutation status and those with known Lynch syndrome and sporadic CRC (Table 2). *BRAF*^*V600E*^ mutation was more frequent in patients with determined Lynch syndrome/sporadic than in those undertermined (39.3% vs. 15.2%) while *RAS* mutation was more observed in patients with undetermined Lynch syndrome (39.3% vs. 26.9%).

**Table 1.** Baseline characteristics of the combined cohort of 466 included patients with MSI-H/dMMR mCRC treated with ICIs.

Characteristics	Total number and percentage
**Sex**	
** Male**	257 (55.2%)
** Female**	209 (44.8%)
**Age at start of ICI therapy, years, mean ± SD (range)**	58.2 ± 14.9 (18.0%-91.0%)
**Tumor sidedness**	
** Right sided**	309 (66.9%)
** Left sided**	153 (33.1%)
**Primary tumor surgery**	423 (90.8%)
***BRAF*** ^**V600E**^	
** Wild type**	319 (68.5%)
** Mutated**	129 (27.7%)
** Undetermined**	18 (3.9%)
***RAS***	
** Wild type**	295 (63.3%)
** Mutated**	153 (32.8%)
** Undetermined**	18 (3.9%)
*** RAS*/*BRAF*** ^**V600E**^ ** wild type**	166 (35.6%)
**Treatment type**	
** Anti-PD1 monotherapy**	305 (65.5%)
** Anti-PD1 + anti-CTL4**	161 (34.5%)
**No. of prior treatment lines**	
** 0**	111 (23.9%)
** ≥1**	354 (76.1%)
**No. of metastatic sites**	
** 1**	196 (42.1%)
** ≥2**	270 (57.9%)
**ECOG performance score**	
** 0**	230 (49.5%)
** 1**	206 (44.3%)
** 2**	29 (6.2%)

Abbreviations: ECOG, Eastern Oncology Cooperative Group; ICI, immune checkpoint inhibitor.

**Table 2.** Baseline characteristics of patients with determined/undermined Lynch syndrome and sporadic CRC.

Characteristics	Total determined Lynch + sporadic (*N* = 242)	Lynch syndrome determined (*n* = 118)	Sporadic (*n* = 124)	Lynch syndrome or sporadic undetermined (*n* = 224)
Sex				
Male	131 (54.1%)	80 (67.8%)	51 (41.1%)	126 (56.3%)
Female	111 (45.9%)	38 (32.2%)	73 (58.9%)	98 (43.8%)
Age at start of ICI therapy, years, median ± SD (range)	58.3 ± 15.0 (21.0%-90.0%)	49.8 ± 12.1 (21.0%-78.0%)	66.4 ± 12.9 (22.0%-90.0%)	58.3 ± 14.7 (18.0%-91.0%)
Tumor sidedness				
Right sided	175 (72.3%)	73 (61.8%)	102 (82.3%)	134 (60.4%)
Left sided	65 (26.9%)	43 (36.4%)	22 (17.7%)	88 (39.6%)
Unknown	2 (0.8%)	2 (1.8%)	0	2 (0.9%)
Primary tumor surgery	218 (90.1%)	106 (89.8%)	112 (90.3%)	205 (91.5%)
Germline *MMR* gene mutation				
* MLH1*	—	29 (24.6%)	—	—
* PMS2*	—	10 (8.5%)	—	—
* MSH2*	—	45 (38.1%)	—	—
* MSH6*	—	20 (16.9%)	–	–
* EPCAM*	—	2 (1.6%)	—	—
Unknown^*^	—	12 (10.1%)	—	—
*RAS*				
Wild type	169 (69.8%)	55 (46.6%)	114 (91.9%)	126 (56.2%)
Mutated	65 (26.9%)	55 (46.6%)	10 (8.1%)	88 (39.3%)
Unknown	8 (3.3%)	8 (6.8%)	0	10 (4.5%)
*BRAF* ^V600E^				
Wild type	139 (57.4%)	109 (92.4%)	30 (24.2%)	180 (80.3%)
Mutated	95 (39.3%)	1 (0.8%)	94 (75.8%)	34^**^ (15.2%)
Unknown	8 (3.3%)	8 (6.8%)	0	10 (4.5%)
*MLH1* hypermethylation	—	—	37	—
Bi-allelic somatic mutations			6	
Treatment type				
Anti-PD1 monotherapy	148 (61.2%)	70 (59.3%)	78 (62.9%)	157 (70.1%)
Anti-PD1 + anti-CTL4	94 (38.8%)	48 (40.7%)	46 (37.1%)	67 (29.9%)
No. of prior treatment lines				
0	58 (24.0%)	28 (23.7%)	30 (24.2%)	53 (23.8%)
≥1	184 (76.0%)	90 (76.3%)	94 (75.8%)	171 (76.2%)
No. of metastatic sites				
1	104 (43.0%)	46 (39.0%)	58 (46.8%)	92 (41.1%)
≥2	138 (57.0%)	72 (61.0%)	66 (53.2%)	132 (58.9%)
ECOG performance score				
0	106 (44.0%)	52 (44.4%)	54 (43.5%)	124 (55.4%)
1	120 (49.8%)	57 (48.7%)	63 (50.8%)	86 (38.4%)
2	15 (6.2%)	8 (6.8%)	7 (5.6%)	14 (6.3%)

^*^Germline mutation identified with missing data for the mutated gene.

^**^Data not available for germinal mutation and MMR immuno-histochemistry (not possible to classify Lynch or sporadic).

Abbreviations: ECOG, Eastern Oncology Cooperative Group; ICI, immune checkpoint inhibitor; MMR, mismatch repair.

### *RAS* and *BRAF*^V600E^ Mutational Status

In the population with known *RAS* and *BRAF*^V600E^ status  (*n* = 448), 194 PFS events were observed. The median  follow-up was 20.9 months. In adjusted analysis of 443 patients with data available for all selected variables (186 events observed), no association with PFS was observed for *BRAF*^*V600E*^ mutation (PFS HR = 1.20, 95% Cl, 0.80-1.79,  *P* = .372) and *RAS* mutation (PFS HR = 0.93, 95% Cl, 0.64-1.36, *P* = .712; Table 3). There were 138 OS events observed. In adjusted analysis with selected variables (133 events observed) (Supplementary Table S1), no association between OS and *BRAF*^*V600E*^ mutation (HR = 1.06, 95% Cl, 0.66-1.70, *P* = .811) and *RAS* mutation (HR =0.75, 95% CI, 0.48-1.17, *P* = .202; Supplementary Table S1) was observed.

**Table 3.** Unadjusted and adjusted hazard ratios for progression-free survival (PFS) in 443 patients with known RAS and BRAF^V600E^ mutational status.^*^

Variable	Unadjusted		Adjusted	
	HR (CI 95%)	*P-*value	HR (CI 95%)	*P*-value
*BRAF* ^*V600E*^ mutated vs. *BRAF*^V600E^ wild type	1.48 (1.10-2.00)	.010	1.20 (0.80-1.79)	.372
*RAS* mutated vs. *RAS* wild type	0.76 (0.55-1.04)	.088	0.93 (0.64-1.36)	.712
Age at start of ICI therapy^**^		.007		.072
48-59 vs. 18-47	1.36 (0.86-2.15)		1.13 (0.70-1.81)	
60-69 vs. 18-47	2.06 (1.34-3.18)		1.76 (1.09-2.85)	
70-91 vs. 18-47	1.72 (1.11-2.67)		1.63 (0.97-2.74)	
Female vs. male	1.09 (0.81-1.45)	.575	0.94 (0.69-1.27)	.680
Left sided vs. right sided	1.01 (0.74-1.37)	.946	1.16 (0.82-1.63)	.403
Anti-PD1 + anti-CTL4 vs. anti-PD1	0.44 (0.31-0.61)	<.001	0.50 (0.34-0.73)	<.001
ECOG performance score		<.001		<.001
1 vs. 0	1.90 (1.40-2.57)		2.01 (1.44-2.81)	
2 vs. 0	3.27 (1.87-5.72)		3.45 (1.84-6.49)	
≥2 metastatic sites vs. 1 metastatic site	1.21 (0.90-1.62)	.219	1.04 (0.76-1.43)	.799
≥1 prior treatment lines vs. 0 prior treatment lines	1.81 (1.20-2.72)	.004	2.09 (1.34-3.27)	.001
Primary tumor surgery vs. no primary tumor surgery	0.76 (0.46-1.26)	.291	0.78 (0.46-1.32)	.351

^*^Only patients with all the data variables available.

^**^The continuous age variable did not meet the proportionality of risk assumption, so it was analyzed in classes defined from quartiles.

Abbreviations: ECOG, Eastern Oncology Cooperative Group; HR, hazard ratio; ICI, immune checkpoint inhibitor.

### Lynch vs. Sporadic

**Figure 3.**
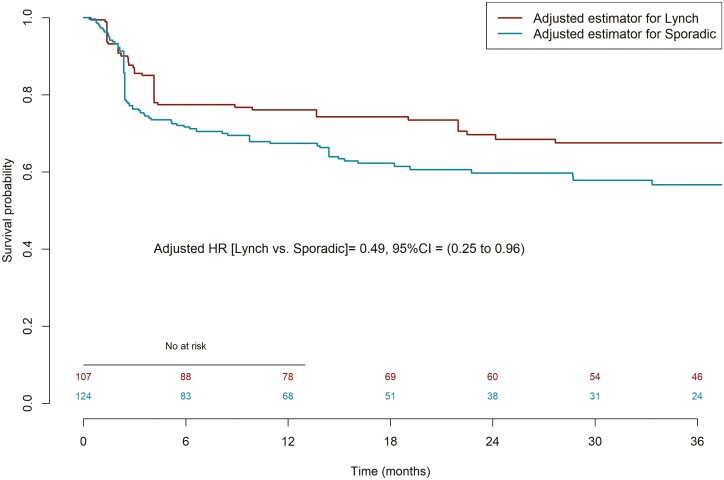
Progression-free survival (PFS) in patients with determined Lynch syndrome and with sporadic MSI-H/dMMR CRC.

In the population with determined Lynch and sporadic status (*n* = 242), 84 PFS events were observed. In unadjusted and adjusted analysis of 231 patients with data available for all selected variables (80 events observed), Lynch syndrome was associated with fewer PFS events compared with sporadic type (HR = 0.40, 95% CI, 0.25-0.64, *P* < .001 and HR = 0.49, 95% CI, 0.25-0.96, *P* = .036, respectively; Table 4 and Fig. 3). The type of germinal mutation in case of Lynch syndrome did not appear to impact the ICIs effect on PFS in the analysis with a limited number of patients (*n* = 104, Supplementary Fig. S1). There were 58 OS events were observed. Adjusted HR for analysis of OS in patients with known Lynch syndrome compared with those with sporadic CRC (54 events observed) was 0.56 (95% CI, HR = 0.25-1.22, *P* = .143; Supplementary Table S2).

**Table 4.** Unadjusted and adjusted hazard ratios for progression-free survival in 231 patients with determined Lynch syndrome and sporadic status.^*^

Variable	Unadjusted		Adjusted	
	HR (CI 95%)	*P*-value	HR (CI 95%)	*P*-value
Lynch vs. sporadic	0.40 (0.25-0.64)	<.001	0.49 (0.25-0.96)	.036
Age at start of ICI therapy	1.03 (1.01-1.04)	<.001	1.01 (0.99-1.03)	.618
Female vs. male	1.43 (0.92-2.22)	.109	1.04 (0.64-1.68)	.877
Left-sided vs. right-sided	0.88 (0.53-1.47)	.627		
Anti-PD1 + anti-CTL4 vs. anti-PD1	0.45 (0.28-0.75)	.002	0.63 (0.36-1.08)	.090
*RAS* mutated + vs. *RAS* wild-type	0.50 (0.28-0.89)	.018	0.86 (0.43-1.73)	.682
ECOG performance score		<.001		.011
1 vs. 0	2.22 (1.35-3.63)		1.92 (1.14-3.23)	
2 vs. 0	4.31 (1.84-10.1)		3.35 (1.35-8.32)	
≥2 Metastatic sites vs. 1 metastatic site	1.06 (0.68-1.67)	.793		
≥1 Prior treatment line vs. no prior treatments lines	1.15 (0.67-1.99)	.612		
Primary tumor surgery vs. no primary tumor surgery	0.56 (0.29-1.08)	.083	0.55 (0.27-1.12)	.101

^*^Only patients with all the data variables available.

Abbreviations: ECOG, Eastern Oncology Cooperative Group; HR, hazard ratio; ICI, immune checkpoint inhibitor.

## Discussion

Our study was undertaken to answer the clinical questions in a cohort of MSI-H/dMMR mCRC patients about the impact of *RAS*/*BRAF*^V600E^ mutational status and Lynch syndrome/sporadic CRC on the efficacy of ICIs. We show that *RAS* and *BRAF*^*V600E*^ mutations do not seem to be molecular modifiers of prognosis in patients who were treated with ICIs. This finding is not in line with PFS data on *RAS* mutation reported from the post-hoc subgroup analysis of the phase III Keynote 177 trial. However, the trial did not determine *RAS* status for all patients (30% of patients had no mutational status data) so this could have led to selection bias.^4^ Nonetheless, previous phase II studies have also highlighted no impact of *RAS* mutation on PFS.^1-3^ Similar results were reported in phase II and III trials subgroups analysis in terms of *BRAF*^*V600E*^ mutation.^1-4^

This work presented a large international cohort study of patients treated for mCRC by ICIs with a strict definition of Lynch syndrome. Our data suggest that Lynch syndrome is protective against PFS events. Lynch syndrome-associated tumors have different clinical, histological, and immunological features, notably higher T cells infiltration, than their sporadic counterparts.^8-10^ Data from the subgroup analyses of previous prospective phase II trials did not support any differences in survival between these 2 groups of patients, but characterization of Lynch syndrome in these studies was done by investigators based on past medical history and other available factors collected from clinical records only without a defined algorithm, which could have led to misclassifications. The classification in the mentioned studies was done by investigators based on past medical history collected from clinical records only or on other available factors.^1-3^ The method used in this international study was based on rigorous classification with the concurrent use of immunochemistry and our designed molecular-based laboratory practice algorithm. Indeed, assigning correctly to Lynch or sporadic MSI-H/dMMR mCRC subgroups demands data, which are not always available or asked in routine practice such as *MLH1* promoter methylation status testing or bi- allelic somatic mutational status analysis in absence of  germline mutation (Fig 2). In our study, we did not find significant differences in OS between Lynch and sporadic groups. A possible reason for this lack of benefit in OS may be the low power to detect significance due to not sufficient valid sample size. However, it should be pointed out that our data are consistent with the literature since the NICHE-2 trial recently showed that preoperative 1-month therapy with ipilimumab and nivolumab achieved an increased pathological complete response rate in patients with Lynch syndrome-associated versus sporadic MSI-H primary colon cancer.^14^ Also, the promising results of organ preservation strategies with 100% clinical complete response in patients with MSI-H rectal cancer may be partially related to the over-representation of Lynch syndrome in patients developing MSI-H cancers in the rectum.^15^ Finally, our data are biologically sound since a previous study demonstrated significantly superior tumor mutational burden in patients with MSH2/MSH6 deficiency and this may lead to increase the immunogenicity of the tumor and, potentially, improved outcomes on immunotherapy, as we showed here.^16^

In spite of the strengths, this study has some limitations. Regarding the analysis of the role of Lynch vs. sporadic cases, a large number of cases were excluded (*n* = 224) due to the absence of molecular data and because they may have potentially biased the selection. The comparison of the population with determined Lynch syndrome/sporadic and the population with excluded cases indicated that there were more *BRAF*^*V600E*^-mutated mCRC in the analyzed population while *RAS* mutation was more frequent in the population with undetermined Lynch syndrome and sporadic CRC (Table 2). This observation is consistent with the fact that *BRAF*^*V600E*^ mutation is a major factor in our algorithm to clearly distinguish Lynch syndrome and sporadic CRC. Although *BRAF*^*V600E*^ mutation was not a prognostic factor in this ICIs-treated MSI-H/dMMR population, it was a selective marker for sporadic cases. We did find that the highest proportion of *BRAF*^*V600E*^ mutation was seen in the sporadic group (75.8%). Moreover, on the molecular level, some misclassifications could persist in our study and in clinical practice due to the marginal phenomenon of germline *MLH1* promoter hypermethylation. Still, the analysis of determined Lynch syndrome/sporadic and excluded cases populations also found that patients matched for clinical prognostic factors, namely age, performance status, number of previous lines for mCRC received, and number of metastatic sites.

These results need to be prospectively validated in subgroup analysis with the addition of Lynch/sporadic cases strictly defined by the algorithm. If doing so, this classification could be used for stratification of MSI-H/dMMR mCRC patients in ICIs-based trials given the absence of a uniform standard.

## Conclusion

This study demonstrated that *RAS and BRAF*^*V600E*^ mutations do not impact prognosis of MSI-H/dMMR patients treated with ICIs. Lynch syndrome-associated CRC might have a better survival compared with sporadic CRC, but this result will require further confirmation studies.

## Supplementary Material

oyad082_suppl_Supplementary_Figure_S1

oyad082_suppl_Supplementary_Tables

## Data Availability

Data may be made available upon request to the corresponding author and upon specific data sharing contract.
