# Supplementary figures and images for: BRAF V600E/RAS Mutations and Lynch Syndrome in Patients With MSI-H/dMMR Metastatic Colorectal Cancer Treated With Immune Checkpoint Inhibitors
Source: Oncologist. 2023 Apr 6;28(9):771–9. doi: 10.1093/oncolo/oyad082 (PMC10485382; doi:10.1093/oncolo/oyad082)

**Figure S1**. Progression-free survival according to germline mutation in Lynch patients


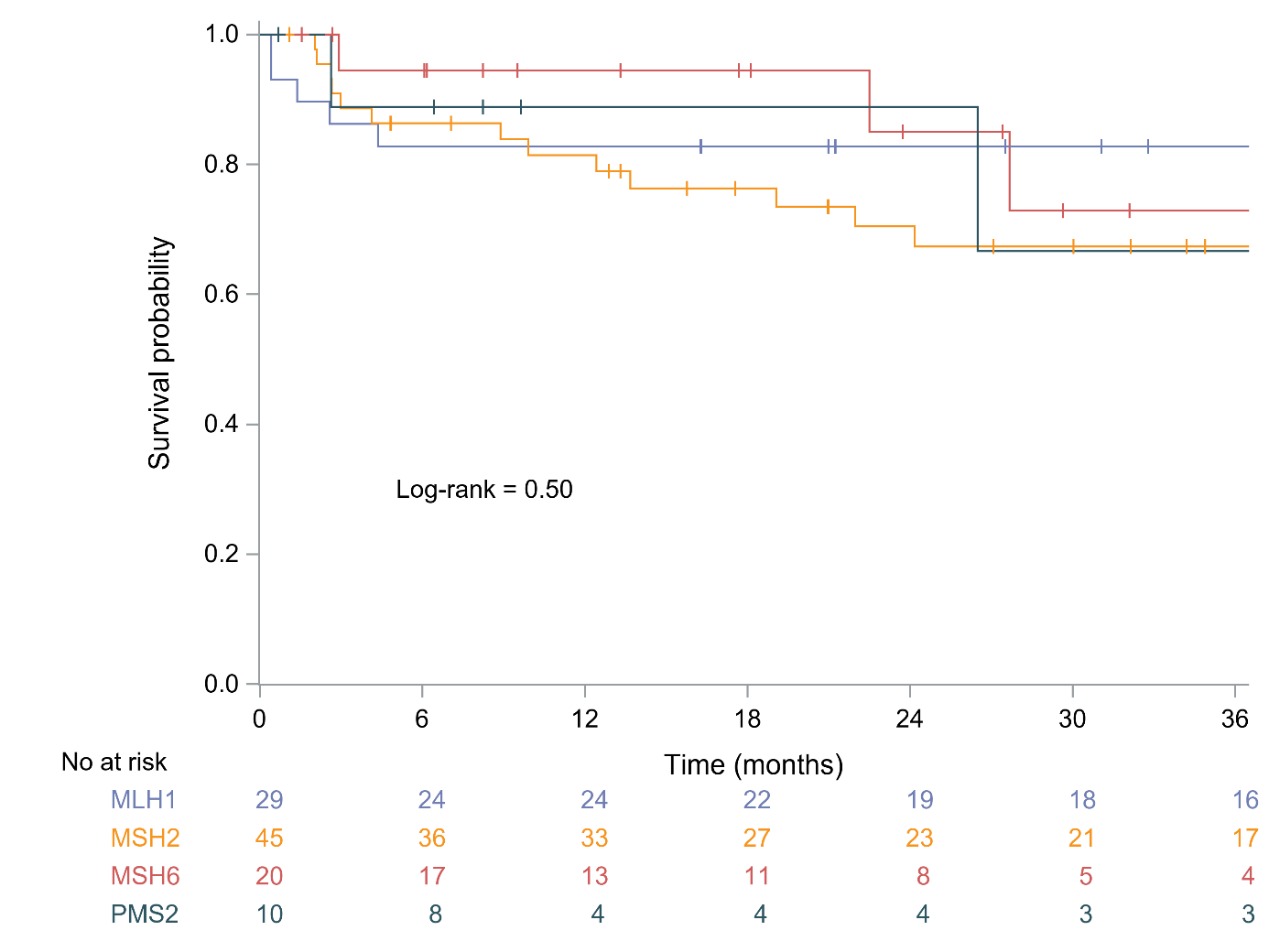

Supplement: oyad082_suppl_Supplementary_Figure_S1 [file oyad082_suppl_supplementary_figure_s1.docx]
