# Supplementary material for: BRAF V600E/RAS Mutations and Lynch Syndrome in Patients With MSI-H/dMMR Metastatic Colorectal Cancer Treated With Immune Checkpoint Inhibitors
Source: Oncologist. 2023 Apr 6;28(9):771–9. doi: 10.1093/oncolo/oyad082 (PMC10485382; doi:10.1093/oncolo/oyad082)
Supplement: oyad082_suppl_Supplementary_Tables [file oyad082_suppl_supplementary_tables.docx]

**Table S1.** Unadjusted and adjusted hazard ratios for OS in 433 patients with known *RAS* and *BRAF*^V600E^ mutational status*

| Variable | Unadjusted | | Adjusted | |
| --- | --- | --- | --- | --- |
|  | HR (CI 95%) | *P* | HR (CI 95%) | *P* |
| *BRAF^V600E^* mutated vs *BRAF* wild-type | 1.36 (0.95 to 1.94) | .092 | 1.06 (0.66 to 1.70) | .811 |
| Age at start of ICI therapy | 1.01 (1.00 to 1.03) | .027 | 1.01 (1.00 to 1.03) | .094 |
| Female vs male | 0.88 (0.63 to 1.25) | .485 | 0.78 (0.54 to 1.12) | .180 |
| Left-sided vs right-sided | 1.24 (0.87 to 1.77) | .230 | 1.32 (0.89 to 1.97) | .165 |
| Anti-PD1 + anti-CL4 vs anti-PD1 | 0.36 (0.24 to 0.56) | <.001 | 0.38 (0.24 to 0.61) | <.001 |
| *KRAS/NRAS* mutated vs *RAS* wild-type | 0.68 (0.46 to 0.99) | .046 | 0.75 (0.48 to 1.17) | .202 |
| ECOG performance score |  | <.001 |  | <.001 |
| 1 vs 0 | 2.01 (1.39 to 2.90) |  | 2.18 (1.45 to 3.25) |  |
| 2 vs 0 | 4.43 (2.39 to 8.21) |  | 4.01 (2.00 to 8.04) |  |
| ≥ 2 metastatic sites vs 1 metastatic site | 1.28 (0.90 to 1.82) | .177 | 1.11 (0.77 to 1.61) | .577 |
| ≥ 1 prior treatment line vs no prior treatment line | 1.98 (1.19 to 3.30) | .008 | 2.44 (1.41 to 4.20) | .001 |
| Primary tumor surgery vs no primary tumor surgery | 0.66 (0.38 to 1.15) | .143 | 0.62 (0.34 to 1.12) | .112 |

^*^ Only patients with all the data variables available CI = confidence Interval; ECOG = Eastern Oncology Cooperative Group; HR = hazard ratio.

**Table S2**. Unadjusted and adjusted hazard ratios for OS in 231 patients with determined Lynch syndrome and sporadic status*

| Variable | Unadjusted | | Adjusted | |
| --- | --- | --- | --- | --- |
|  | HR (CI 95%) | *P* | HR (CI 95%) | *P* |
| Lynch vs sporadic | 0.38 (0.21 to 0.69) | .001 | 0.56 (0.25 to 1.22) | .143 |
| Age at strt of ICI therapy | 1.03 (1.01 to 1.05) | .002 | 1.01 (0.99 to 1.03) | .421 |
| Female vs male | 1.39 (0.82 to 2.38) | .225 |  |  |
| Left-sided vs right-sided | 1.02 (0.55 to 1.87) | .954 |  |  |
| Dual therapy vs monotherapy | 0.37 (0.20 to 0.69) | .002 | 0.43 (0.22 to 0.84) | .014 |
| *RAS* mutated vs *RAS* wild-type | 0.41 (0.19 to 0.87) | .021 | 0.74 (0.30 to 1.82) | .514 |
| ECOG performance score |  | <.001 |  | .003 |
| 1 vs 0 | 2.42 (1.30 to 4.51) |  | 2.57 (1.29 to 5.08) |  |
| 2 vs 0 | 6.82 (2.59 to 18.0) |  | 5.26 (1.83 to 15.1) |  |
| ≥2 metastatic sites vs 1 metastatic site | 0.96 (0.56 to 1.65) | .878 |  |  |
| ≥1 prior treatment line vs no prior treatment line | 1.18 (0.59 to 2.35) | .634 |  |  |
| Primary tumor surgery vs no primary tumor surgery | 0.50 (0.23 to 1.11) | .089 | 0.47 (0.20 to 1.10) | .081 |

^*^ Only patients with all the data variables available. CI = confidence Interval; ECOG = Eastern Oncology Cooperative Group; HR = hazard ratio.
